# Supplementary material for: Metabolically Activated Proteostasis Regulators Reduce Differentiation of CD4+ TH17 Cells
Source: bioRxiv. 2026 Sep 5:2026.09.02.747634. Preprint. [Version 1] doi: 10.64898/2026.09.02.747634 (PMC13684497; doi:10.64898/2026.09.02.747634)
Supplement: Supplementary file 1 [file NIHPP2026.09.02.747634V1-supplement-1.pdf]

bioRxiv preprint doi: https://doi.org/10.64898/2026.09.02.747634; this version posted September 5, 2026. The copyright holder for this preprint (which was not certified by peer review) is the author/funder, who has granted bioRxiv a license to display the preprint in perpetuity. It is made available under a CC-BY 4.0 International license.

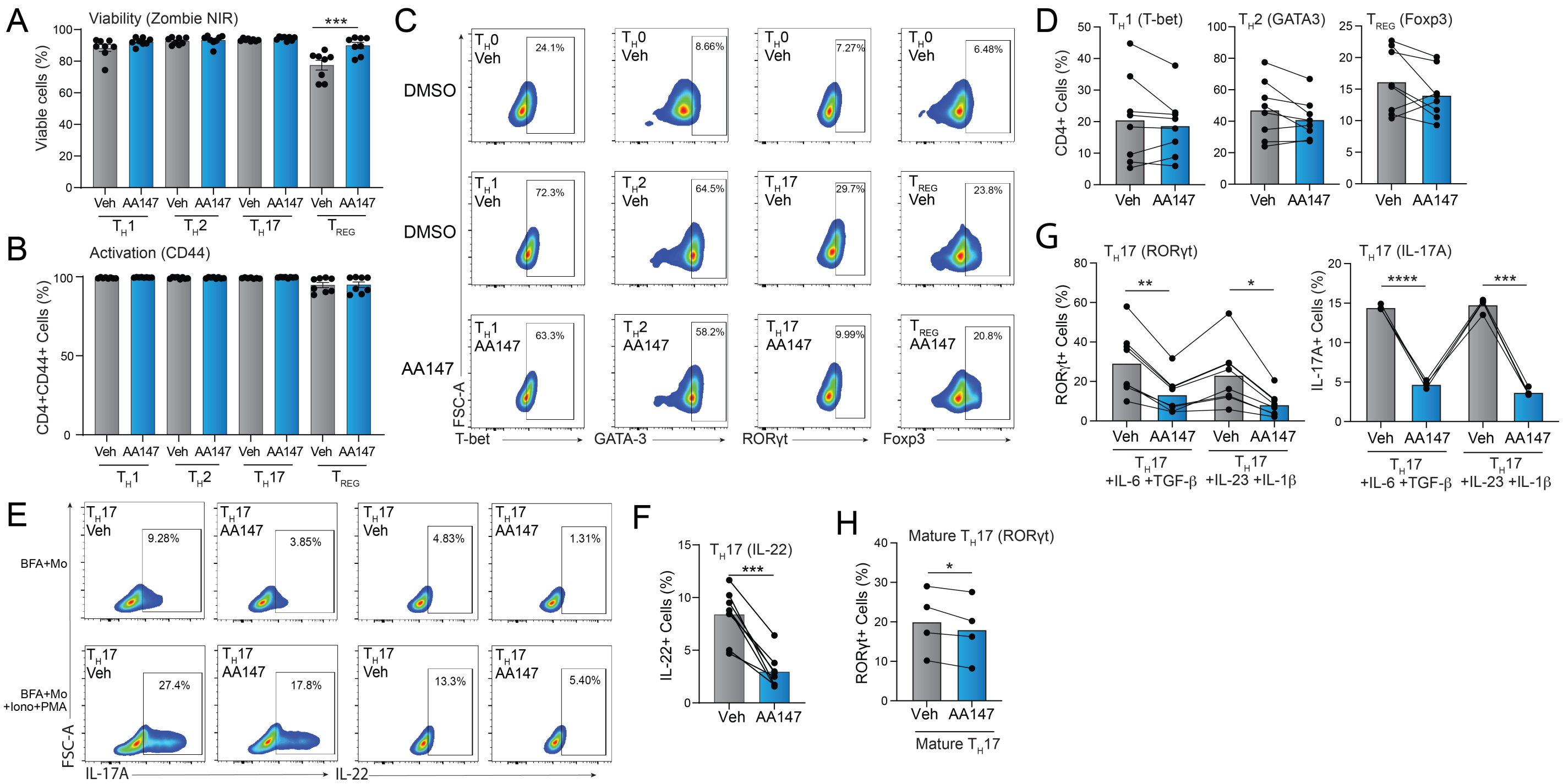

Figure S1 (Supplement to Figure 1). AA147 preferentially decreases differentiation of CD4$^+$ T_H17 cells. A,B. Viability, measured by Zombie NIR, (**A**), and activation, measured by CD44 staining (**B**), of CD4$^+$ T cells differentiated for 72 h in T_H1, T_H2, T_H17 or T_{REG} polarising conditions in the absence or presence of AA147 (30 μM). **C.** Representative flow cytometry images of the indicated lineage-defining transcription factors in activated CD4$^+$ T cells differentiated for 72 h in T_H1 (T-bet), T_H2 (GATA3), T_H17 (RORγt), or T_{REG} (Foxp3) polarising conditions in the presence or absence of AA147. **D**. Percentage of CD4$^+$ T-bet$^+$ (T_H1, left), CD4$^+$ GATA3$^+$ (T_H2, middle), or CD4$^+$ Foxp3$^+$ ($T_{REG,}$ right) T cells differentiated in the presence of vehicle or AA147 (30 μM) for 72 h. Data are shown for CD4$^+$ naïve T cells isolated from n=8 mice. **E.** Representative flow cytometry images for CD4$^+$ IL-17A$^+$ (left) and CD4$^+$ IL-22$^+$ (right) T cells differentiated for 72 h in T_H17 polarising conditions and then rested for 2 h followed by re-stimulation for 3 h by treatment with phorbol 12-myristate 13-acetate (PMA, 50 ng/mL), ionomycin (Iono, 1 μg/mL), brefeldin A (BFA, 1 μg/mL), and monensin (Mo, 1 μg/mL). Unstimulated cells were rested for 2 h followed by treatment for 3 h with BFA (1 μg/mL) and Mo (1 μg/mL) are shown as a control. **F**. Percentage of CD4$^+$ IL-22$^+$ T cells differentiated in the presence of vehicle or AA147 (30 μM) for 72 h. Data are shown for CD4$^+$ naïve T cells isolated from n=8 mice. **G**. Percentage of CD4$^+$ RORγt$^+$ (left) or CD4$^+$ IL-17A$^+$ (right) T cells differentiated for 72 h in the indicated T_H17 polarising cytokine combination in the absence or presence of AA147 (30 μM). Data are shown for CD4$^+$ naïve T cells isolated from n=8 mice. **H**. Percentage of CD4$^+$ RORγt$^+$ T cells differentiated for 72 h in T_H17 polarising conditions and then treated for an additional 72 h with AA147 (30 μM). Data are shown for CD4$^+$ T cells isolated from n=4 mice. *$p<0.05$, **$p<0.01$, ***$p<0.005$, ****$p<0.001$ for one-way ANOVA (panels **A**, **G**) or paired t-test (panels **D, F**, **H**). Data described in panels A, B, D, F, G, and H are provided in **Source Data Figure S1.**

bioRxiv preprint doi: https://doi.org/10.64898/2026.09.02.747634; this version posted September 5, 2026. The copyright holder for this preprint (which was not certified by peer review) is the author/funder, who has granted bioRxiv a license to display the preprint in perpetuity. It is made available under a CC-BY 4.0 International license.

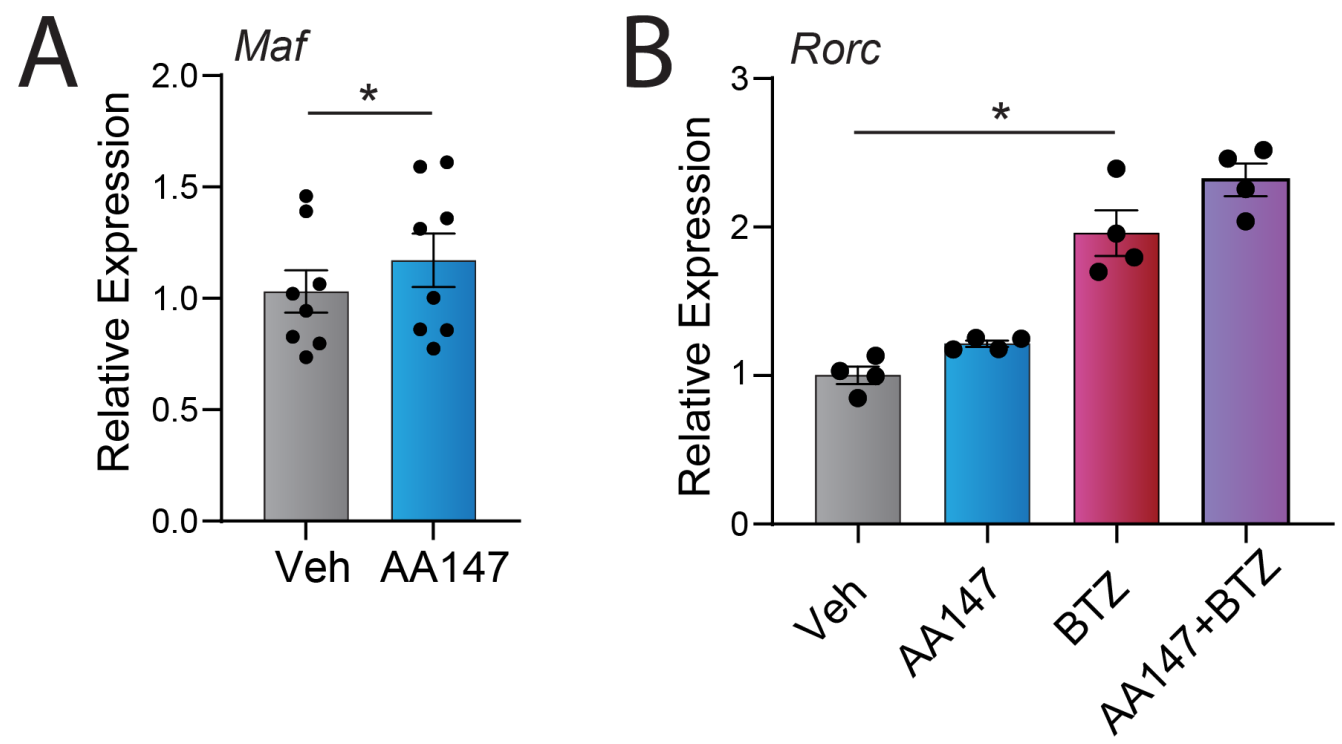

Figure S2 (Supplement to Figure 2). AA147 promotes proteasomal degradation of RORγt. A. Expression, measured by RT-qPCR, of the pSTAT3 target gene *Maf* in CD4⁺ T cells differentiated for 72 h in T_H17 polarising conditions in the presence or absence of AA147 (30 μM). Data are shown for CD4⁺ T cells isolated from n=8 mice. **B**. Expression, measured by RT-qPCR, of *Rorc* in CD4⁺ T cells differentiated for 72 h in T_H17 polarising conditions in the presence or absence of AA147 (30 μM), followed by treatment with vehicle or BTZ (10 μM) for 4 h, as indicated. Data are shown for CD4⁺ T cells isolated from n=4 mice. *$p<0.05$ for paired t-test (panel **A**) or one-way ANOVA (panel **B**). Data described in panels A and B are provided in **Source Data Figure S2.**

bioRxiv preprint doi: https://doi.org/10.64898/2026.09.02.747634; this version posted September 5, 2026. The copyright holder for this preprint (which was not certified by peer review) is the author/funder, who has granted bioRxiv a license to display the preprint in perpetuity. It is made available under a CC-BY 4.0 International license.

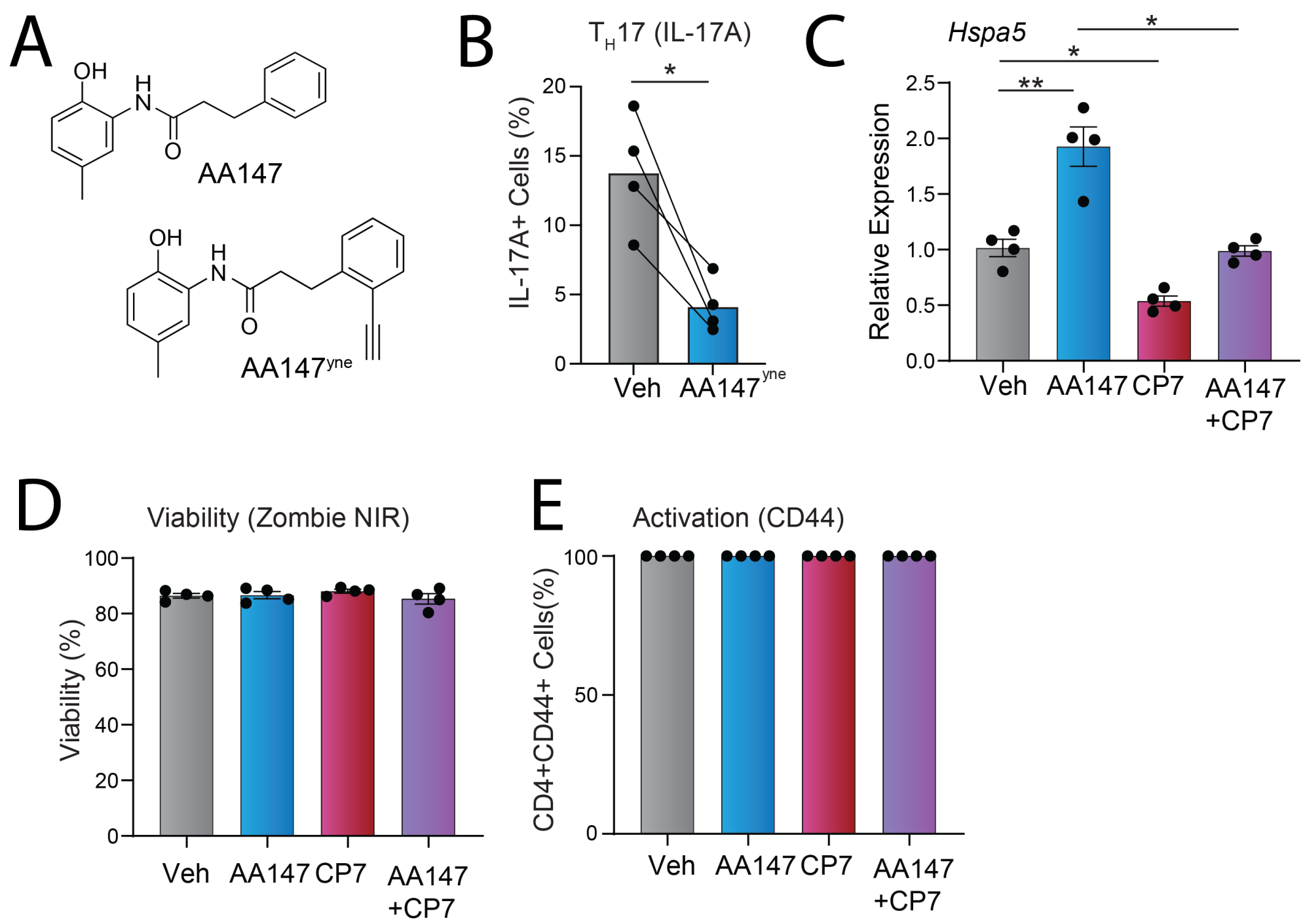

Figure S3 (Supplement to Figure 3). AA147-dependent reductions in T_H17 differentiation are independent of ATF6 activation. A. Structure of AA147 and the alkyne functionalised probe $AA147^{yne}$. **B.** Percentage of $CD4^+$ IL-$17A^+$ T_H17 cells differentiated for 72 h in the presence of $AA147^{yne}$ (30 µM). Data are shown for CD4+ T cells isolated from n=4 different mice. **C.** Expression, measured by RT-qPCR, of *Hspa5* in $CD4^+$ T cells differentiated for 72 h in T_H17 polarising conditions in the presence or absence of AA147 (30 µM) and/or CP7 (3 µM). **D, E.** Viability, measured by Zombie NIR (**D**), and activation, measured by CD44 staining (**E**), of $CD4^+$ T_H17 cells differentiated for 72 h in the absence or presence of AA147 (30 µM) and/or CP7 (3 µM), as indicated. Data are shown for $CD4^+$ T cells isolated from 4 different mice. *p<0.05, **p<0.01, for one-way ANOVA (panel **C, D, E**) or paired t-test (panel **B**). Data described in panels B-E are provided in **Source Data Figure S3.**

bioRxiv preprint doi: https://doi.org/10.64898/2026.09.02.747634; this version posted September 5, 2026. The copyright holder for this preprint (which was not certified by peer review) is the author/funder, who has granted bioRxiv a license to display the preprint in perpetuity. It is made available under a CC-BY 4.0 International license.

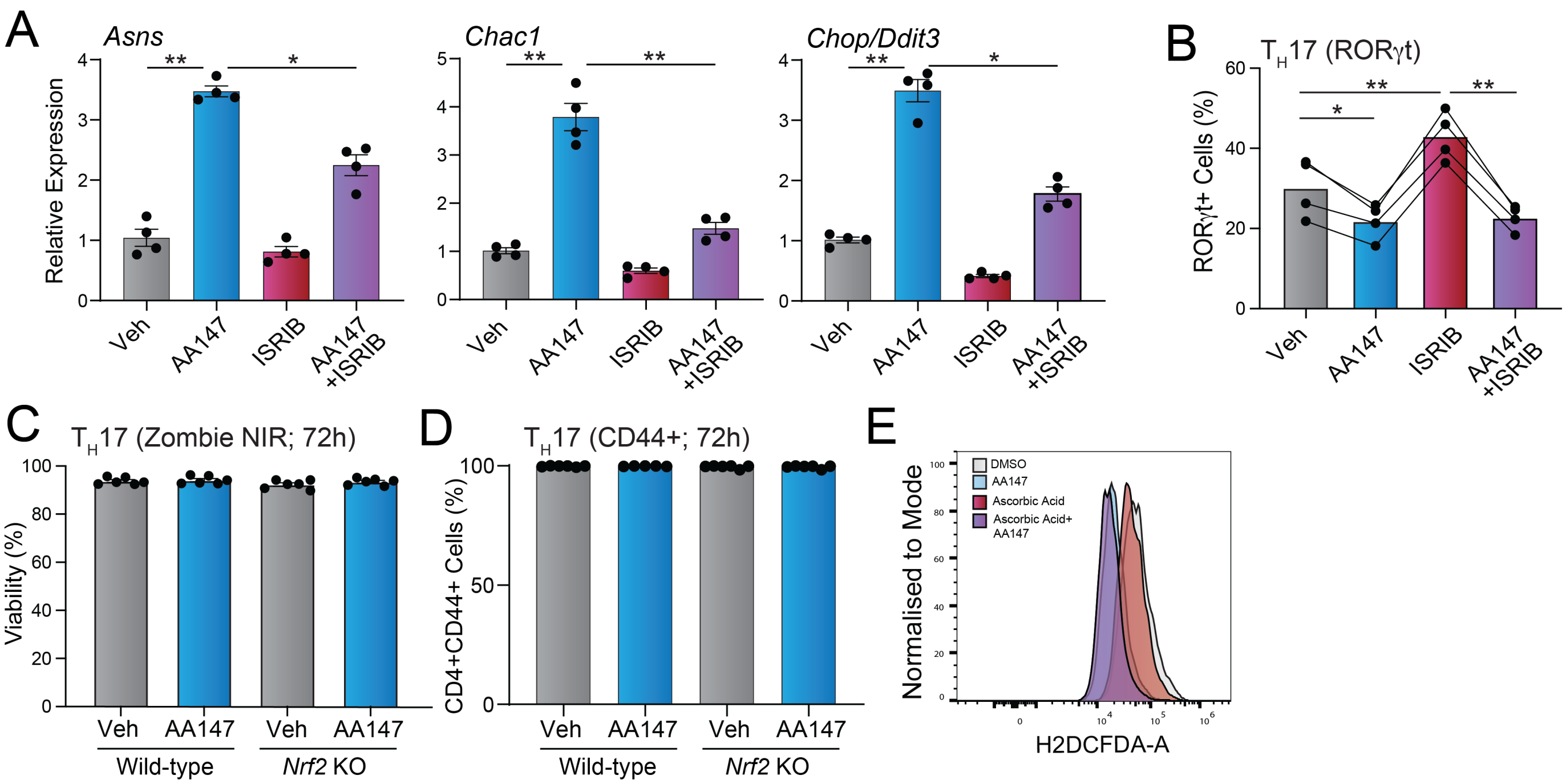

Figure S4 (Supplement to Figure 4). AA147 reduces T_H17 differentiation through an NRF2-dependent mechanism. A. Expression, measured by RT-qPCR, of the ISR target genes *Asns* (left), *Chac1* (middle) or *Chop/Ddit3* (right) in $CD4^+$ T cells differentiated for 72 h in T_H17 polarising conditions in the presence or absence of AA147 (30 μM) and/or ISRIB (250 nM). Data are shown for $CD4^+$ T cells isolated from n=4 mice. **B.** Percentage of $CD4^+$ $ROR\gamma t^+$ T_H17 cells differentiated for 72 h in the presence or absence of AA147 (30 μM) and/or ISRIB (250 nM). Data for $CD4^+$ T cells isolated from n=4 mice are shown. **C, D.** Viability, measured by Zombie NIR, (**C**), and activation, measured by CD44 staining (**D**), of $CD4^+$ T cells differentiated from wild-type or *Nrf2* KO mice for 72 h in the T_H17 polarising conditions in the absence or presence of AA147 (30 μM). Data for $CD4^+$ T cells isolated from n=4 mice are shown. **E.** Representative flow cytometry histograms of H_2DCFDA fluorescence in T_H17 cells differentiated for 72 h in the presence or absence of AA147 (30 μM) and/or ascorbic acid (250 μM). *p<0.05, **p<0.01, for one-way ANOVA (panels **A**, **B, C, D**). Data described in panels A-D are provided in **Source Data Figure S4.**

bioRxiv preprint doi: https://doi.org/10.64898/2026.09.02.747634; this version posted September 5, 2026. The copyright holder for this preprint (which was not certified by peer review) is the author/funder, who has granted bioRxiv a license to display the preprint in perpetuity. It is made available under a CC-BY 4.0 International license.

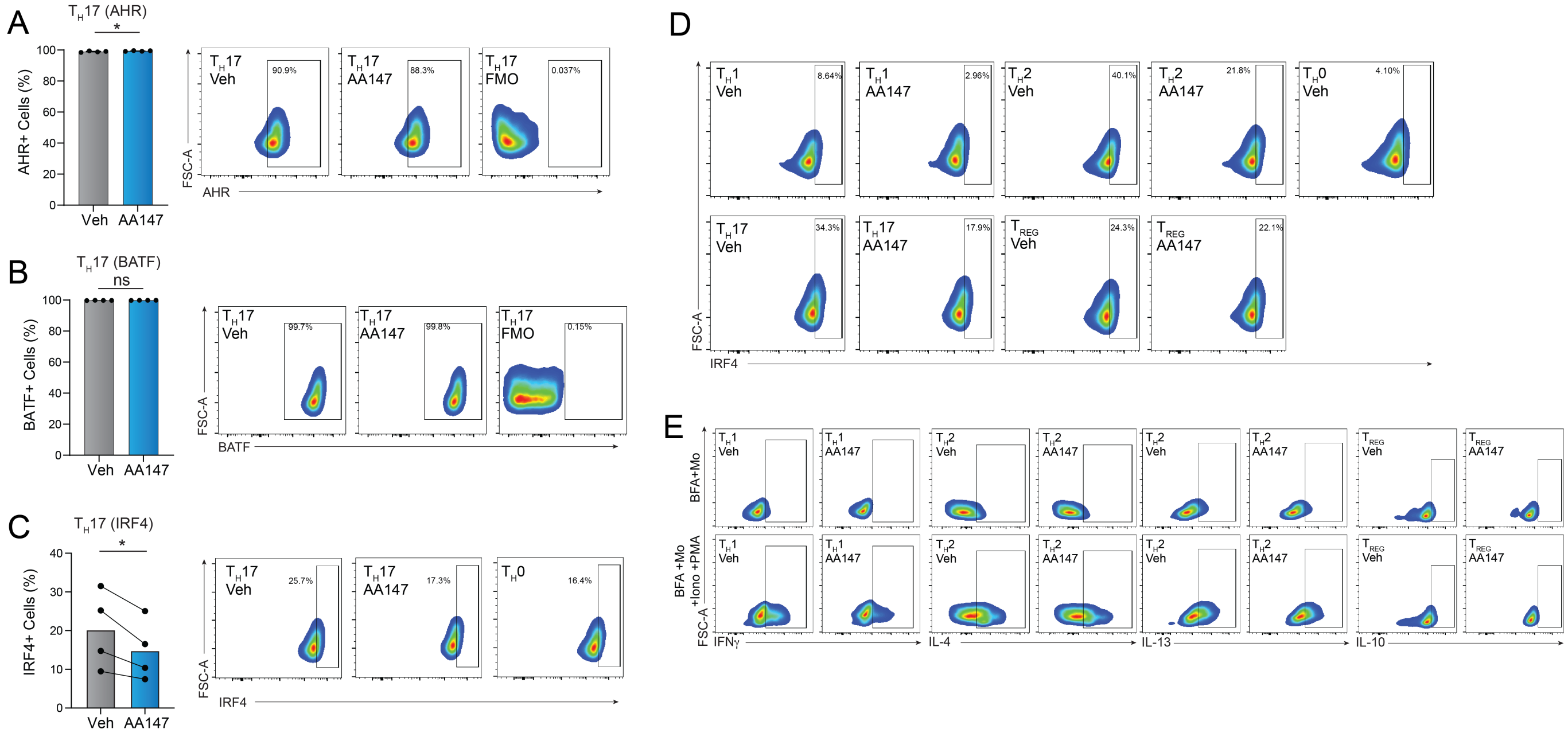

Figure S5 (Supplement to Figure 5). AA147 reduces IRF4 in CD4⁺ T cells through an NRF2-independent mechanism. A-C. Representative flow cytometry plot and quantification of $CD4^+$ AHR^+, $CD4^+$ $BATF^+$, or $CD4^+$ $IRF4^+$ cells differentiated for 72 h in T_H17 polarising conditions in the presence or absence of AA147. **D**. Representative flow plots of $CD4^+$ IRF4+ in cells differentiated for 72 h in T_H1, T_H2, T_H17, or T_{REG} polarising conditions, as indicated, in the presence or absence of AA147 (30 μM). **E.** Representative flow plots of $CD4^+$ $IFN\gamma^+$, $IL\text{-}4^+$, $IL\text{-}13^+$, or $IL\text{-}10^+$ cells differentiated for 72 h in T_H1, T_H2, or T_{REG} polarising conditions, as indicated, in the presence or absence of AA147 (30 μM). ***p<0.005, ****p<0.001 for paired t-test (panels **A, B, C**). Data described in panels A-C are provided in **Source Data Figure S5.**

bioRxiv preprint doi: https://doi.org/10.64898/2026.09.02.747634; this version posted September 5, 2026. The copyright holder for this preprint (which was not certified by peer review) is the author/funder, who has granted bioRxiv a license to display the preprint in perpetuity. It is made available under a CC-BY 4.0 International license.

SUPPLEMENTAL TABLE LEGENDS

Table S1: TMT-MS proteomic data from T_H17 cells differentiated in the presence or absence of AA147 (30 μM) for 72 h. Data shown as relative fold changes with corresponding p values. A p value cut off of 0.05 was used.

Table S2: RNAseq data from TH17 cells differentiated in the presence or absence of AA147 (30 μM) for 72 h. A p value cut off of 0.05 was used.

Table S3: RNAseq data from TH17 cells differentiated in the presence or absence of AA147 (30 μM) for 72 h showing genes used in the *Rorc* gene list as well as the stress pathway profiling gene list.

SOURCE DATA FILES

Source Data Figure 1. Source data used to generate **Figure 1B, D, E**.

Source Data Figure S1. Source data used to generate **Figure S1A, B, D, F, F, G, H**.

Source Data Figure 2. Source data used to generate **Figure 2B-E**.

Source Data Figure S2. Source data used to generate **Figure S2A, B**.

Source Data Figure 3. Source data used to generate **Figure 3C-F**.

Source Data Figure S3. Source data used to generate **Figure S3B-E**.

Source Data Figure 4. Source data used to generate **Figure 4C-I**.

Source Data Figure S4. Source data used to generate **Figure S4-D**.

Source Data Figure 5. Source data used to generate **Figure 5A-F**.

Source Data Figure S5. Source data used to generate **Figure S5A-C**.
